# Safety Evaluation of the Coagulase-Negative Staphylococci Microbiota of Salami: Superantigenic Toxin Production and Antimicrobial Resistance

**DOI:** 10.1155/2015/483548

**Published:** 2015-11-30

**Authors:** Raquel Soares Casaes Nunes, Eduardo Mere Del Aguila, Vânia Margaret Flosi Paschoalin

**Affiliations:** Instituto de Química, Universidade Federal do Rio de Janeiro, Avenida Athos da Silveira Ramos 149, Sala 545, Cidade Universitária, 21949-909 Rio de Janeiro, RJ, Brazil

## Abstract

The risks of contracting staphylococci food poisoning by the consumption of improperly manufactured salami and the possibility of this food being reservoirs for antibiotic resistance were evaluated. Nineteen coagulase-negative staphylococci (CNS) strains were found in commercial and artisanal salami. The species in commercial salami were* S. saprophyticus*,* S. sciuri*,* S. xylosus*, and* S. carnosus*. Artisanal salami showed* S. succinus*,* S. epidermidis*, and* S. hominis* but no* S. carnosus*. Phylogenetic analyses grouped the strains into three major staphylococcal species groups, comprised of 4 refined clusters with similarities superior to 90%. Fifteen strains harbored multiple enterotoxin genes, with high incidence of* seb*/*sec* and* sea*, 57% and 50%, respectively, intermediate incidence of* sed*/*seh*/*selm* and* sei*/*seln*/*tst*-H, 33% and 27%, correspondingly, and low incidence of* see*/*selj*/*selo* and* seg*, of respectively 13% and 1%. Real time RT-PCR and enzyme-linked-immunosorbent assays confirmed the enterotoxigenicity of the strains, which expressed and produced enterotoxins* in vitro*. The CNS strains showed multiresistance to several antimicrobials of therapeutic importance in both human and veterinarian medicine, such as *β*-lactams, vancomycin, and linezolid. The effective control of undue staphylococci in fermented meat products should be adopted to prevent or limit the risk of food poisoning and the spread of antimicrobial-resistant strains.

## 1. Introduction

Staphylococcal food poisoning is an illness caused by the ingestion of contaminated food containing enterotoxins produced by bacteria belonging to this genus. Enterotoxins that exhibit superantigenic activities are heat stable proteins and may not be destroyed even during cooking conditions.

In Brazil, according to data from the Ministério da Saúde (Ministry of Health), staphylococcal poisoning is the second most common foodborne disease, ranking only after outbreaks involving* Salmonella *spp. [1].* Staphylococcus* classified as coagulase-positive are considered potential food enterotoxin-producing strains [1], although, recently, the enterotoxigenic potential of coagulase-negative staphylococci (CNS) species in food poisoning has also been recognized [2].

Initially, enterotoxin SEs family members were divided into five serological types (*sea* through* see*) based on their antigenicity [3, 4]. In recent years, however, newly described types of SEs—SEG, SEH, SEI, SElJ, SElK, SElL, SElM, SElN, SElO, SElP, SElQ, SElR, and SElU—with amino acid sequences similar to the classical SEs, were discovered. These newly described enterotoxins are designated as SE or SE-like (SEl), according to their emetic properties displayed in a primate model following oral administration [5].

The toxic shock syndrome toxin-1 (TSST-1) is also a member of the SE-related toxin family and has the ability to stimulate large populations of T cells containing a particular V*β* element in their T-cell receptors (TCR). Like other superantigenic toxins, it bypasses normal antigen presentation by binding to class II major histocompatibility complex molecules on antigen-presenting cells and to specific variable regions on the beta-chain of the T-cell antigen receptor. Through this interaction, a massive proliferation of T cells at orders of magnitude above antigen-specific activation occurs, resulting in a massive cytokine release that is believed to be responsible for the most severe features of TSST [6].

Enterotoxin (SE) genes are encoded in mobile genetic elements, such as plasmids, prophages, and* Staphylococcus* pathogenic islands (SaPIs) [7].

Salami is a kind of dry sausage obtained by the microbial fermentation of raw pork meat, using Staphylococcus* starter cultures* as technological accessories to ferment the product and give it its organoleptic characteristics. In Brazil, Italian type salami, similar to salami produced in Southern Europe, is avidly consumed, with a production trade of 13.093 tons between 2000 and 2014 [8].

*S. xylosus*,* S. equorum*, and* S. carnosus* are part of the starter culture microbiota that participate in the reactions required for creating the flavor and aroma during the maturing period of fermented meat production [9]. In addition, other species, such as* S. epidermidis*,* S. pasteuri*,* S. sciuri*, and* S. succinus*, may also occasionally be present in meaningful amounts [10].

However, even the combination of physical and chemical barriers cannot always guarantee the stability and microbial safety of starter cultures. Contamination of salami fermentation starter culture microbiota by pathogenic coagulase-negative staphylococci (CNS) strains is perhaps the most harmful factor in the production of cured meat products, since these pathogens are able to produce heat-stable enterotoxins with superantigenic activities in food matrices [11, 12].

Staphylococci species are not usually identified at the species level by routine laboratory testing and commercial kits, since phenotypic discrimination cannot reliably identify these species due to the variable expression of some phenotypic traits [13]. For this purpose, molecular techniques, including nucleotide sequencing within the* 16S rDNA*,* hsp60*,* tuf*,* sodA*, and* rpoB* genes, have been successfully used to identify* Staphylococcus* species [14].

Depending on the conditions, some species of coagulase-negative staphylococci can present health risks, since they have shown resistance to several antibiotics of therapeutic importance, such as *β*-lactams [15].

The aim of the present study was to identify the members of the CNS microbiota from salami. We sampled the salami marketed in Brazil, comparing the CNS microbiota in salami produced by industrial companies and in artisanal salami manufactured by small producers. The CNS strains were identified by sequencing of a 16S rDNA region and the phylogenic relationships between the observed species were established. The presence of multiple genes encoding the classical and newly described* se*/*sel* and* tstH1* toxins in the CNS genomes was investigated. The risk of food poisoning was assessed by evaluating the ability of the CNS strains in transcribing and expressing the classical and newly described enterotoxins* in vitro* by using real time RT-PCR and enzyme-linked immunosorbent assay (ELISA). The resistance of the isolated strains to antimicrobial agents of therapeutic importance in staphylococci infections was also evaluated.

## 2. Materials and Methods

### 2.1. Isolation of Bacterial Strains

Six samples of distinct brands of salami, 03 from the meat industry and 03 from small artisanal producers, were collected in the municipality of Rio de Janeiro, Brazil. Twenty-five grams of salami was added to 225 mL of 0.1% peptone water. The suspensions were transferred to homogenizer bags (Interscience, Saint Nom, France) and coupled to a Stomacher^@^ 400 circulator (Seward, Worthing, West Sussex, UK) at 260 rpm for 1 min. The suspensions were serial-diluted from 10^−6^ to 10^0^ and 100 *μ*L of each dilution was transferred onto 20 *μ*L of Baird-Parker agar containing egg yolk tellurite emulsion (BPAþ RPF, bioMerieux, France). Eighty presumptive coagulase-negative staphylococci colonies were tested by Gram-staining, catalase, coagulase, and thermostable DNAse activities according to Bergey's Manual of Systematic Bacteriology. Sixty presumptive CNS strains were stored at −80°C in tryptone soy agar (TSA, Franklin Lakes, New Jersey, USA) plus 45% v/v glycerol.

### 2.2. DNA Preparation

The strains were cultured aerobically overnight in 10 mL Brain Heart Infusion broth (BD BBL, Le Pont de Claix, France) at 37°C for 24 h. The suggestive CNS colonies were adjusted to 10^6^ UFC/mL in a spectrophotometer and harvested by centrifugation at 5,700 × g for 1 min. The cell pellet was used for DNA extraction using the DNeasy blood and tissue kit (Qiagen, Dusseldorf, Germany), following the manufacturer's instructions. Genomic DNA was quantified using the Qubit fluorometer (Invitrogen, Grand Island, New York, USA) and Qubit assay kits.

### 2.3. PCR Tests 

#### 2.3.1. Primer Sequences and Target Genes

Primer sets flanking the* sea*,* seb*,* sec*,* sed*,* see*,* seg*,* seh*,* sei*,* selk*,* selm*,* seln*,* selo*,* selq*,* selr*,* selu*, and* tstH1* sequences are listed in Table 1.

#### 2.3.2. Uniplex-, Duplex-, and Multiplex-PCR Tests

Uniplex-PCR tests targeting the* tstH1* sequence and duplex-PCR targeting the* sea/seb* and* sec/sed* sequences were performed. PCR mixtures contained 25 *μ*L of 20 mM MgCl_2_, 10x PCR buffer (Invitrogen, Grand Island, New York, USA), 100 mM dNTP mix (Fermentas Thermo Scientific, Vilnius, Lithuania), 0.2 mM of each primer (Table 1), 0.5 U Taq DNA polymerase (Invitrogen, Grand Island, New York, USA), and 100 ng of DNA templates. Uniplex- and duplex-PCR assays were performed under the following conditions: 94°C for 5 min followed by 35 cycles of 94°C for 2 min, 53°C for 2 min, and 72°C for 1 min for extension, ending with a final extension at 72°C after 7 min [16], with modifications in the annealing temperature, using a thermal cycler (MyCycler, Bio-Rad, Hercules, CA, USA). The amplified fragments were visualized on 1.0% agarose gels (Sigma) stained with GelRed (dilution 1 : 1000) (BioAmerica, Tel Aviv, Israel) and documented on a transilluminator (MiniLumi Imaging Bio-Systems, BioAmerica, Tel Aviv, Israel).

#### 2.3.3. Multiplex-PCR Tests

Multiplex-PCR assays were performed by the simultaneous amplification of the* see*,* seg*,* seh*,* sei*,* selj*,* selm*,* seln*,* selo*,* selk*,* selq*,* selr*, and* selu* sequences using the primer sets listed in Table 1. Each reaction contained 50 *μ*L of a mix containing 0.5 U Taq DNA polymerase, 10x PCR buffer, 100 mM dNTP, 0.2 *μ*M of each primer, and 100 ng of DNA template. DNA amplification of* see*,* seg*,* seh*, and* sei* was carried out as follows: 95°C for 5 min, 35 cycles of 95°C for 30 s, 53°C for 90s and 72°C for 90 s, and a final extension at 72°C for 10 min. The DNA amplifications of the* selj, selm, seln,* and* selo* group and the* selk, selq, selr, *and* selu* group were carried out in the same conditions [3]. PCR products were visualized by electrophoresis on 1.2% agarose gels (Uniscience do Brasil, São Paulo, Brazil) in 1x TAE (Tris-boric acid-EDTA) buffer stained by 0.5 *μ*g mL^−1^ of GelRed (BioAmerica, Tel Aviv, Israel) and documented on a transilluminator (MiniLumi Imaging Bio-Systems, BioAmerica, Tel Aviv, Israel).

DNA templates from the following reference strains were used:* S. aureus* ATCC 29231 (*sea*),* S. aureus* ATCC 14458 (*seb, tstH, selk, selq, selr,* and* selu*);* S. aureus* ATCC 19095 (*sec, seg, seh,* and* sei*),* S. aureus* ATCC 13563 (*sed*),* S. aureus* ATCC 27664 (*see*), and* S. aureus* ATCC 27154 (*selj, selm, seln,* and* selo*) and* S. xylosus* ATCC 29971.

### 2.4. Enterotoxin Expression Assays

The observed strains were cultured aerobically overnight in 10 mL Brain Heart Infusion Broth (BD BBL, Le Pont de Claix, France) at 37°C for 72 h. Bacteria supernatants were collected by centrifugation at 4,000 ×g for 10 min and used for the detection of* sea, seb, sec, sed*, and* see* by an ELISA assay using a commercial detection kit (RIDASCREEN SET A, B, C, D, E Art. number R4101, R-Biopharm AG, Germany). The assay was performed according to the manufacturer's recommendation and as described elsewhere [16]. The mean lower limit of detection of the assay was 0.25 ng mL^−1^. The threshold is defined as the average OD of two negative controls plus 0.15, a constant established by the kit. Samples containing SEs showed absorbance values equal to or greater than the threshold value. All experiments were performed in duplicate.

### 2.5. Real Time RT-PCR Assays

Total RNA was extracted by using the QIAGEN RiboPure Bacteria kit (Life Technologies, Carlsbad, California, USA) following the manufacturer's instructions and quantified using the Qubit fluorometer (Invitrogen, Grand Island, New York, USA) and Qubit assay kits. The cDNA synthesis was performed by using the High Capacity cDNA Reverse Transcription Kit (Applied Biosystems, California, USA) and the ABI PRISM 7500 Fast RT-PCR system (Applied Biosystems, California, USA). Samples were plated in triplicate in 96-well plates as follows: 12 *μ*L of the SYBR Green PCR Master Mix; 1 *μ*L of primer mix (*sea, seb, sec, sed,* and* see*); and 4.5 *μ*L of the cDNA ultrapure water in each well. Amplification was performed under the following conditions: 95°C for 15 min, 40 cycles at 95°C for 15 s, 54°C for 30s, and 72°C for 30 s. The dissociation curve was performed at 95°C for 15 sec, 54°C for 30 sec, and 95°C for 15 sec. CT means, the standard deviations, and the cDNA semiquantification were calculated using the GraphPad Prism 5 software package. Calibration curves based on five points were constructed in triplicate corresponding to serial dilutions (1, 1 : 10, 1 : 100, 1 : 1000, and 1 : 10000) from 100 ng of a DNA template stock solution.

### 2.6. 16S rDNA Sequencing

Amplification of the V5 region of 16S rDNA fragment was performed using 50 ng of DNA templates from the 65 strains found in the salami samples. PCR was performed under the following conditions: 95°C for 10 min, followed by 30 cycles at 95°C for 30 s, 60°C for 30 s and 72°C for 45 s, and a final extension at 72°C for 10 min. PCR products were purified using the PCR DNA Purification Kit (Applied Biosystems, California, USA) and sequenced using 20 ng purified DNA and 13 *μ*L of primer sets in a final volume of 20 *μ*L. After amplification, products were purified according to the protocol of the BigDye Terminator Purification X Kit (Applied Biosystems, California, USA) and sequenced on a 3130 sequencer Genetic Analyzer (Applied Biosystems, California, USA). The sequences were compared to the 16S rDNA gene sequences of* Staphylococcus* species available at the GenBank database (http://www.ncbi.nlm.nih.gov/Genbank/index.html). Multiple sequence alignments were performed using Clustal W (Kyoto University, Bioinformatics Center; http://www.genome.jp/tools/clustalw/).

### 2.7. Sequencing of Enterotoxin PCR Products

PCR products were purified using the PCR DNA Purification Kit (Applied Biosystems, California, USA) and sequenced using 10 ng of purified DNA and 3.2 pmoles of each primer set in a final volume of 20 *μ*L. After amplification in the same conditions as the PCR step (Section 2.3.3), products were purified according to the BigDye Terminator Purification X Kit protocol (Applied Biosystems, California, USA) and sequenced on a 3130 sequencer Genetic Analyzer (Applied Biosystems, California, USA). The sequences were compared to* Staphylococcus aureus* and* Staphylococcus pasteuri* gene sequences available at the GenBank database (http://www.ncbi.nlm.nih.gov/Genbank/index.html). Multiple sequence alignments were performed using Clustal W (Kyoto University, Bioinformatics Center; http://www.genome.jp/tools/clustalw/).

### 2.8. Phylogenetic Analyses

Phylogenetic relationships between the CNS strains were performed by sequence alignments using the Clustal X 2.0 software package [24]. The phylogenetic trees were constructed using the software Mega 6.0 and UPGMA methods [25].

### 2.9. Antibiotic Susceptibility Tests

An inoculum of each strain equivalent to a 0.5 McFarland scale was swabbed onto a Mueller-Hinton agar plate (BD BBL Franklin Lakes, New Jersey, USA**)** and the antibiotic disc was then placed on the plate followed by overnight incubation at 37°C. The inhibition zone was interpreted according to the Clinical Laboratory Standard (CLSI) Guidelines, formerly known as the National Committee for Clinical Laboratory Standards. The tested antibiotics were penicillin G (10 U), oxacillin (1 *μ*g), neomycin (30 *μ*g), sulfamethoprim (5 *μ*g), clindamycin (2 *μ*g), gentamicin (10 *μ*g), cefoxitin (30 *μ*g), rifampicin (5 *μ*g), erythromycin (15 *μ*g), tetracycline (30 *μ*g), vancomycin (30 *μ*g), ciprofloxacin (5 *μ*g), sulfazothrim (23 *μ*g), cefepime (30 *μ*g), linezolid (30 *μ*g), and chloramphenicol (30 *μ*g).

### 2.10. Minimal Inhibitory Concentration (MIC) Determinations

The MICs of vancomycin, linezolid, methicillin, and ampicillin were determined by the macrodilution broth method based on CLSI recommendations, using in-house-prepared panels [26]. Antibiotic concentrations of 0.03, 0.06, 0.125, 0.25, 0.5, 1.0, and 2.0 mg mL^−1^ were tested. One mL of broth was transferred to the tubes and 100 *μ*L of the bacteria suspension was adjusted to 10^6^ CFU/mL in saline 0.85% according to a 0.5 McFarland scale and transferred to tubes containing 1 mL of each antimicrobial. Strains were grown in Mueller-Hinton broth (BD BBL Franklin Lakes, New Jersey, USA) and the MIC was estimated as the lowest antibiotic concentration that inhibits visible growth after 24 h [26].

## 3. Results and Discussion

### 3.1. Isolation and Identification of Coagulase-Negative Strains from Salami

Sixty-five presumable coagulase-negative staphylococci microorganisms from salami were isolated by colony morphology, coagulase slide test, subsequent tube test, and biochemical tests. The sequencing of the V5 region of the 16S rDNA fragment of the strains was discriminative enough to differentiate the* Staphylococcus* isolated from salami at the subspecies level, with the exception of 1 strain, identified up to the* Staphylococcus* spp. genus.

Nineteen distinct strains were identified as CNS in salami with 08 of 19 (42%) identified as* S. saprophyticus*, the predominant species, followed by 05 strains of* S. xylosus* (26%), 02 strains of* S. carnosus* (11%), and 01 strain of each of the following species:* S. succinus*,* S. sciuri*,* S. epidermidis*, and* S. hominis* (5% each) (Table 2).

The 08* S. saprophyticus* strains were identified as KJ699151.1, AB697717.1, JX490122.1, KJ004623.1, and HQ699510.1, EU430992.1, HF937252.1, and KJ949606.1, with the latter two and* S. sciuri* JX966436.1 being homologous (96–98%) to strains from the environment. Five* S. xylosus* strains, CP007208.1, KF198080.1, CP008724.1, AM882700.1, and KC456590.1, and a single* S. succinus* strain, KC329824.1, were identified. The last three* S. xylosus* strains and the* S. succinus* strain are homologous (97-98%) to strains found in fermented meat or meat starter cultures.* S. xylosus* CP007208.1 showed homology (98%) to potential opportunistic pathogenic strains from mammal species.

The* S. carnosus* strains identified were KJ862002.1 and NR116434.1, and the latter, as well as the single* S. hominis* JX519988.1 (96-97%) identified, were homologous (97%) to species from human microbiota.

*S. carnosus* and* S. xylosus* are commonly used as commercial starter cultures for sausage manufacturing [26], but* S. succinus* has also been observed in dry fermented sausages and its use as a* starter* culture has been already proposed [27].

The diversity of CNS microbiota found in the samples analyzed in the present study could be related to the origin of the salami. The microbiota from the artisanal salami showed greater biodiversity when compared to the commercial salami. In commercial salami,* S. saprophyticus* was the predominant species, but* S. xylosus and S. carnosus*, also observed in commercial salami, are commonly isolated from starter cultures [26]. The predominance of* S. saprophyticus* followed by* S. xylosus* has also been reported in salami from South Italy [28], similar to Belgian sausages, where* S. saprophyticus* was the most frequently detected species [29].

*S. carnosus* was not observed in artisanal salami, but* S. succinus*,* S. epidermidis,* and* S. hominis* were detected.

The biodiversity of the CNS staphylococci species found in microbiota depends on the kind of meat-fermented product, but CNS strains such as* S. saprophyticus*,* S. auricularis*,* S. xylosus*,* S. capitis*,* S. hominis*,* S. carnosus*,* S. haemolyticus*,* S. warneri*,* S. equorum*,* S. cohnii*,* S. capitis*, and* S. intermedius* have been described in Napoli-type salami, Sremska sausages, dry sausages, raw meat, and naturally fermented meat [30–33].

During the last decades* S. epidermidis* and* S. saprophyticus* have been described as emerging pathogens [34].* S. saprophyticus* is considered a frequent contaminant of fermented sausages and raw meats and has been isolated from rectal swabs of cattle carcasses and pigs. In humans, the main reservoir of* S. saprophyticus* is the gastrointestinal tract [35].* S. saprophyticus* and* S. epidermidis* can be opportunistic pathogens, isolated from the human urinary tract, and the presence of these species in food should be taken into account concerning possible contamination of the starter inoculum and/or improvements in the salami manufacturing process [13].

Other CNS species observed in the present study are mainly associated with ordinary food contaminants, with* S. epidermidis* and* S. hominis* being the dominant species in human skin and occasionally isolated from the skin of domestic animals [36].

### 3.2. Phylogenetic Relationships of the CNS Identified in Salami

**Figure 1:**
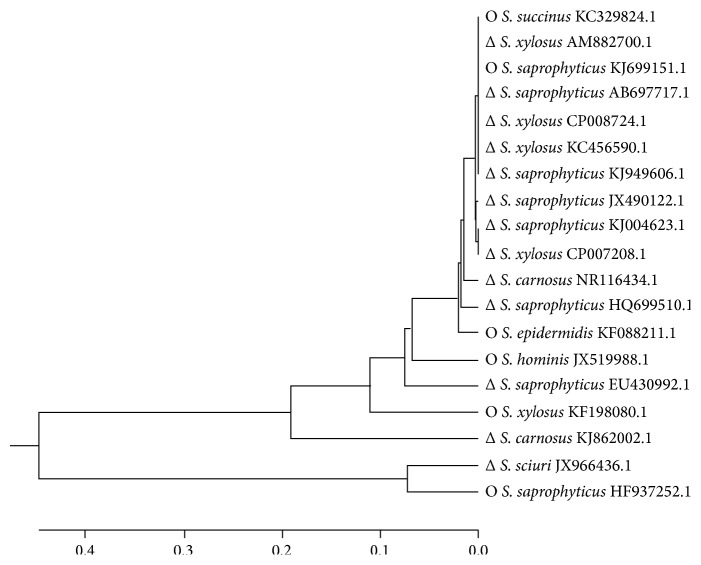
Phylogenetic tree generated from the multiple alignments of the 16S rDNA sequences of CNS strains found in salami using the ClustalX 2.0 software. The phylogenetic tree was constructed by using the Mega 6.0 software and the unweighted pair group method (UPGMA). Bootstrap values ranged from 0.0 to 0.4. Strains found in commercial (Δ) or artisanal salami (O).

CNS strains can be grouped into four species groups:* saprophyticus*,* simulans*,* epidermidis*, and* haemolyticus.* Frequently, the* saprophyticus* species group includes* S. xylosus* and* S. saprophyticus*, while the* simulans* species group is comprised of* S. carnosus* and* S. piscifermentans*; the* epidermidis* species group is composed of* S. epidermidis, S. capitis, S. caprae, *and* S. saccharolyticus* and the* haemolyticus* species group encompasses* S. haemolyticus, S. hominis,* and* S. devriesei* [37, 38]. In the present study, the main cluster grouped several* S. saprophyticus* strains, namely, KJ699151.1, AB697517.1, KJ949606.1, JX490122.1, and KJ004623.1, four* S. xylosus* strains CP008724.1, CP007208.1. AM882700.1, and KC456590.1, and the* S. succinus* KC329824.1 strain (Figure 1). These strains are homologous to those from fermented meat microbiota.

The subclusters of those clusters showed mismatches of species belonging to the four species groups. The first subcluster grouped* S. epidermidis* HF088211.1,* S. saprophyticus* HQ699510.1, and* S. carnosus* NR116434.1 and the second subcluster grouped* S. hominis* JX519988.1 and* the S. saprophyticus* EU430992.1, all of them originally isolated from animals and human beings. The third subcluster includes species previously isolated from the marine environment, such as* S. xylosus* KF198080.1, while* S. carnosus* KJ862002.1 is utilized as a probiotic organism in foods. The fourth cluster grouped the* S. sciuri* JX966436.1 and* S. saprophyticus* HF937252.1 strains, which are homologous to species found in soil.

The CNS strains clustered into groups near the bottom of the phylogenetic tree are mostly strains found in artisanal salami, whereas the species at the top of the tree are mainly CNS strains found in commercial salami (Figure 1).

The close similarities between the* S. saprophyticus* AB697517.1 and KJ949606.1 strains, the* S. succinus* KC329824.1,* S. xylosus* AM882700.1, KC456590.1, and CP008724.1 strains, the* S. saprophyticus* KJ004623.1 and KJ699151.1 strains, and* S. xylosus* CP007208.1 are supported by a bootstrap value of 100%. The interspecies similarities were over 90%, which demonstrates close phylogenies between these CNS strains. Suzuki et al., 2012, demonstrated a bootstrap value higher than 90% for interspecies similarities between* S. saprophyticus, S. epidermidis, S. hominis,* and* S. carnosus*.

### 3.3. Genotypic and Phenotypic Characterization of CNS Strains

Twelve distinct combinations of staphylococcal enterotoxins genes were found in the 15 CNS strains, comprising SEs A–E, G–J, and also the enterotoxin-like toxins (SElL) K–R and U (Table 2).

**Figure 2:**
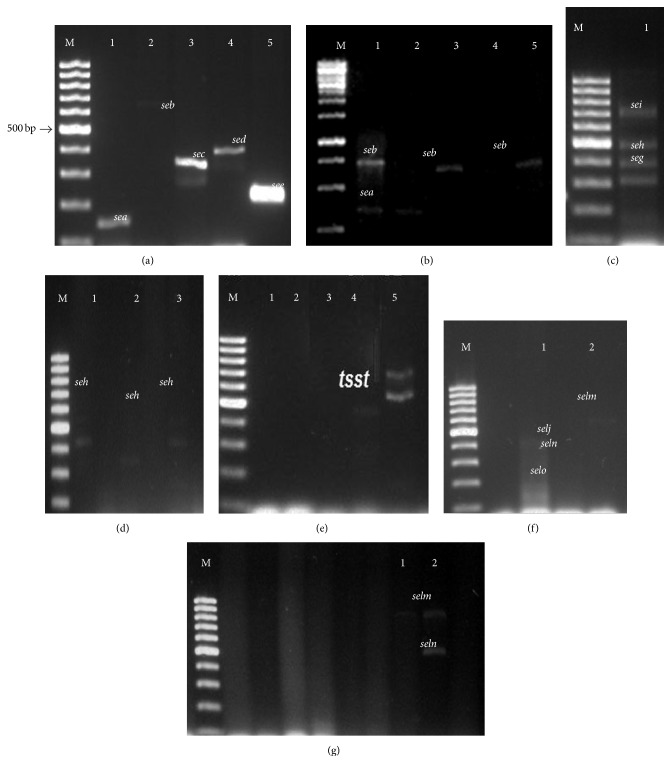
Uniplex, duplex, and multiplex PCR screening for the detection of enterotoxin genes in CNS strains from salami. (a) Lane M, 100 bp DNA ladder plus (Fermentas, Foster City, CA, USA); lane 1,* S. aureus* ATCC 29231 harboring* sea* gene; lane 2,* S. aureus* NCTC 10654 harboring* seb* gene; lane 3,* S. aureus* ATCC19095 harboring the* sec* gene; lane 4,* S. aureus* ATCC 13563 harboring the* sed* gene; and lane 5,* S. aureus* ATCC 27664 harboring the* see* gene. (b) Lane M 100 bp DNA ladder plus; lane 1,* Staphylococcus* spp.; lane 2,* S. carnosus *NR116434; lane 3,* S. carnosus* KJ862002; lane 4,* S. carnosus* KJ862002; and lane 5,* S. carnosus* KJ862002.1. (c) Lane M, 100 bp DNA ladder plus; lane 1,* S. aureus* ATCC 19095 harboring* seg*,* seh*, and* sei* genes. (d) Lane M, 100 bp DNA ladder plus; lane 1,* S. saprophyticus* AB697717.1; lane 2,* S. epidermidis* KF 600589.1; and lane 3,* S. sciuri* JX966436.1. (e) Lane M, 100 bp DNA ladder plus; lane 1,* S. xylosus* KF198080.1; lane 2,* S. saprophyticus* KJ699151.1. (f) Lane M, 100 bp DNA ladder plus; lane 1,* S. aureus* ATCC 27154 harboring* selj*,* slem*,* seln*, and* selo* genes. (g) Lane M, 100 bp DNA ladder plus; lane 1,* S. xylosus* KF198080.1; and lane 2,* S. saprophyticus* subsp.* bovis* KJ699151.1.

Fifteen strains (79%) carried at least one gene encoding enterotoxins in their genomes (Figure 2). The* seb* and* sec* genes were the most predominant, harbored by 57% of the strains, followed by* sea,* carried by 50%, whereas the* sed/seh/selm* genes showed intermediate incidence harbored by 33% of the strains, while* sei/seln* and* tsH1* were found in 27% of strains. Finally,* see, selj,* and* selo* showed low incidence (13%) and* seg* was carried by only 1% of strains (Table 2).

The relationships between superantigenic toxin genotypes and toxin gene-encoding mobile genetic elements in CNS strains were evaluated. Distinct combinations of* SaPI* and plasmids or plasmids and genes of* egc* operon enterotoxins were found in the CNS strains obtained from salami.

Four strains presented only the classical enterotoxin genes, namely,* S. saprophyticus* KJ004623.1 and HF937252.1,* S. xylosus* CP008724.1, and* S. carnosus* NR116434.1. Another strain,* S. saprophyticus* AB697717.1, presented only the newly described enterotoxins* seh, sei, *and* selm* and, finally, 10 strains presented a combination of classical and newly described enterotoxin genes in their genomes, with at least one of each enterotoxin type (Table 2).

Previous studies have shown that* sea* is the most common toxin associated with* Staphylococcus* food poisoning, followed by* sed* and* see*, with SEH and SEI being considered as playing only a minor role [39]. Among the 15 enterotoxigenic strains found in salami, 73% of them harbored at least the* sea* gene or combinations of these 05 genes. Strains* S. carnosus* KJ862002.1, found in commercial salami, and* S. epidermidis* KF600589.1, found in artisanal salami, carry 04 of these genes. The* sea* and* seb* enterotoxin genes are known to occupy the same locus on the chromosome, which may explain why these enterotoxins are commonly found together in food poisoning outbreaks [36]. The combination of* sea* and* seb* genes was found in 05 strains:* S. saprophyticus* EU430992.1,* S. carnosus* KJ862002.1,* S. hominis* JX519988.1,* S. saprophyticus* KJ949606.1, and* S. succinus* KC329824.1. A single strain,* S. carnosus* KJ862002.1, showed the combination of* sea* and* sei* genes.

Additionally, some CNS are able to produce TSST-1 alone or in combination with other enterotoxins. Herein, 04 of the 19 strains (21%),* S. saprophyticus* EU430992.1*, S. saprophyticus *subsp.* bovis* KJ699151.1, and* S. xylosus* CP008724.1 and KF198080.1, were shown to harbor the* tstH1* combined with* se* and/or* sel* enterotoxin genes (Table 2).

The staphylococci enterotoxins genes* seg* and* sei* [40] are part of a chromosomal operon gene cluster (*egc*), comprising five genes designated as* selo*,* selm*,* sei*,* seln,* and* seg*. Two of the CNS strains determined in the present study,* S. saprophyticus* EU430992.1 and* S. hominis* JX519988.1, were shown to carry the* selm, seln,* and* selo* genes and the* selm* and* seln* sequences were detected in the genome of a single strain,* S. saprophyticus* subsp.* bovis* KJ699151.1.* S. epidermidis* KF600589.1 showed a combination of* seg* and* sei* genes and 03 strains harbored a single gene,* sei or selm or seln*, perhaps due to the high degree of genetic polymorphism in the chromosomal assembly [41]. Indrawattana et al., 2013, detected the* seg-sei-selm-seln-selo* of the highly prevalent* egc* locus was 26.1% in contrast to this study where 13% CNS presents the combination of* selm, seln,* and* selo* genes [42].

The CNS strains clustered into groups near the bottom of the phylogenetic tree carried the classical enterotoxin genes, while the species at the top of the tree showed high diversity among the enterotoxin genes, combining the classical and the newly described genes in their genomes (Figure 1).

To assess the risk of staphylococcal food poisoning, the ability of the identified strains in harboring the* se* and* sel* genes and in expressing and producing enterotoxins* in vitro* was evaluated. The mRNA for each enterotoxin gene was evaluated by real time RT-PCR assays and enterotoxin content was estimated by a sandwich enzyme immunoassay for the combined detection of* Staphylococcus* enterotoxins (SET) A, B, C, D, and E.

The fifteen enterotoxigenic CNS strains were able to express the classical enterotoxins SEA,* seb*,* sec*,* sed*, and* see*, in concentrations ranging from 0.3 ng mL^−1^ to 1.4 ng mL^−1^, as assessed by the* in vitro* assays (Table 2). The* S. saprophyticus* AB697717.1, KJ699151.1, JX490122.1, KJ004623.1, and HF937252.1 strains,* S. xylosus* KF198080.1, and* S. carnosus* NR116434.1 produced low amount of enterotoxins, lower than 0.5 ng mL^−1^.* S. epidermidis* KF600589.1,* S. saprophyticus bovis* KJ699151.1, and* S. hominis* JX519988.1 produced intermediate amount of enterotoxins, ranging from 0.7 to 0.90 ng mL^−1^, while* S. sciuri* JX966436.1,* S. saprophyticus* EU430992.1,* S. carnosus* KJ862002.1,* S. xylosus* CP008724.1, and* S. succinus* KC329824.1 produced enterotoxins in concentrations ≥1.0 ng mL^−1^.

Although the sandwich enzyme immunoassay is considered the most sensitive method to detect* sea*–*see* enterotoxins, able to detect 0.125 ng mL^−1^, differences in the specificity and sensitivity of the assays for the detection of staphylococcal enterotoxins from foods are expected [43]. A single strain,* Staphylococcus* spp. KF135445.1, which harbors both the* sea* and* seb* genes, was unable to produce* sea*–*see* enterotoxins.

The mRNA levels evaluated by the real time RT-PCR assays for enterotoxins were detected in 12 of 15 strains (86%) (Table 2). Transcripts for the classical enterotoxins genes were detected when* S. saprophyticus* HF937252.1,* S. carnosus* NR116434.1,* S. xylosus* CP008724.1, and* S. succinus* KC329824.1 were assayed. Transcripts for newly described enterotoxins were observed in* S. saprophyticus* JX490122.1 and AB697717.1,* S. sciuri* JX966436.1,* S. saprophyticus *subsp.* bovis* KJ699151.1, and* S. hominis* JX519988.1. Transcripts from both the classical and newly described enterotoxins were detected in* S. saprophyticus* EU430992.1 and KJ949606.1 and* S. epidermidis* KF600589.1.

No mRNA transcripts were obtained for* S. saprophyticus* KJ0046232.1 and* S. carnosus* KJ862002.1, although enterotoxin production was detected by the enzyme-linked immunosorbent tests (Table 2).

The 04 strains that carry* tstH1* in their genome,* S. saprophyticus* EU430992.1 and KJ699151.1, and* S. xylosus* CP008724.1 and KF98080.1 were not able to produce mRNA for TSST-1 in the assay conditions.

The production of classical enterotoxins* in vitro* (immunologic test) matched the results shown by real time RT-PCR assays for the following strains:* S. saprophyticus* JX490122.1,* S. sciuri* JX966436.1,* S. saprophyticus* EU430992.1,* S. saprophyticus* HF937252.1,* S. saprophyticus *subsp.* bovis* KJ699151.1,* S. hominis* JX519988.1,* S. epidermidis* KF600589.1, and* S. succinus* KC329824.1.

Ten strains, namely,* S. saprophyticus* AB697717.1,* S. saprophyticus* EU430992.1,* S. saprophyticus* HF937252.1,* S. saprophyticus* KJ699151.1*, S. saprophyticus* KJ949606.1,* S. succinus KC329824.1*,* S. hominis* JX519988.1,* S. xylosus* KF198080.1 and* S. xylosus* CP008724.1, and* S. epidermidis* KF600589.1 expressed mRNA for multiple* se* and/or* sel* genes. There is a differential transcription among these genes, where the most frequent among the classical ones were* seb* and* see/sea*, transcribed by 04 and 02 strains, respectively, and the most frequent among the newly described genes were* sei* and* seh/seln/selo,* transcribed by 03 and 02 strains, respectively.

No mRNA for the* sec* gene was detected, although* S. saprophyticus* AB697717.1 was able to produce the enterotoxin* in vitro*, as detected by the ELISA assays.

### 3.4. Enterotoxin Gene Homologies between Salami CNS and CPS Strains

**Figure 3:**
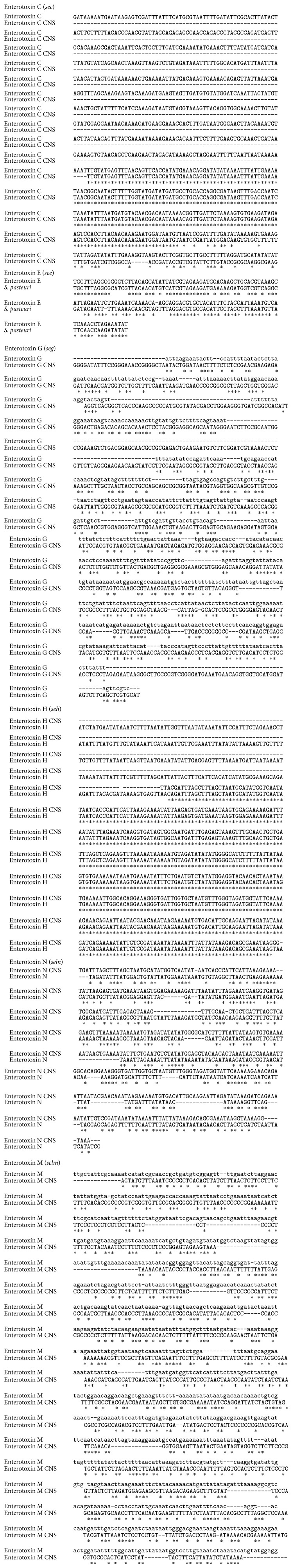
Alignment of the enterotoxin gene sequences found in coagulase-negative staphylococci (CNS) and in coagulase-positive staphylococci (CPS).

The nucleotide sequencing of six enterotoxin genes—two of them,* sec* and* see,* encoding classical enterotoxins—and four of them, encoding the newly described genes,* seg, seh, selm,* and* seln* from CNS strains, was compared to the enterotoxin genes from* S. aureus* or* S. pasteuri*, two coagulase-positive staphylococci strains (Figure 3). The homology between the CNS and CPS enterotoxin genes varied from 65% to 98%. The homology of* sec, seg, seh, selm,* and* seln* between CNS from salami and* S. aureus* was 98%, 60%, 98%, 65%, and 70%, respectively. The homology between* see* from CNS found in salami and in* S. pasteuri* was 98%.

**Figure 4:**
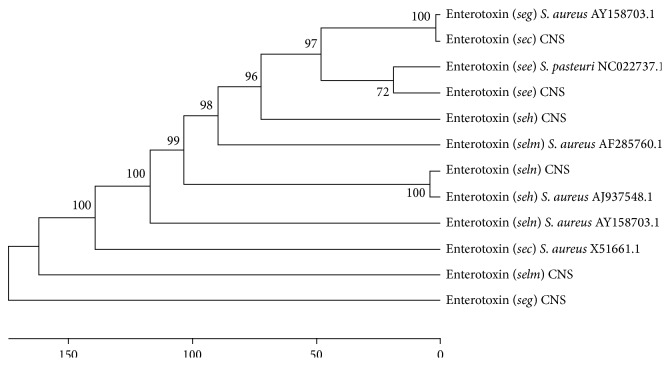
Phylogenetic tree generated from the multiple alignments of the enterotoxin sequences of CNS and CPS strains using the ClustalX 2.0 software package. The phylogenetic tree was constructed using the Mega 6.0 software and the unweighted pair group method (UPGMA).

Although further studies should be performed, it seems that the sequences encoding enterotoxins can be conserved among coagulase-negative and coagulase-positive staphylococci, as shown in the phylogenetic analysis of the 06 enterotoxin genes (Figure 4).

### 3.5. Antimicrobial Multiresistance of CNS Strains (MRCNS)

Another safety hazard associated with CNS strains besides the ability of producing enterotoxins in food matrices is the antimicrobial resistance to antimicrobial agents commonly used to treat staphylococci infections. The antimicrobial resistance carried by CNS strains from food matrices can be spread to the population by the consumption of an apparently safe food.

Among the 19 CNS strains identified in salami, 14 showed multiresistance to antimicrobial agents. Three strains showed the highest MAR indices, 0.93 and 0.80; 07 strains presented MAR indices varying from 0.66 to 0.46, and the remaining 04 strains showed MAR indices ≤0.26 (Table 3).

The 14 MRCNS strains were resistant to *β*-lactams (oxacillin, penicillin, and/or cefoxitin) and to vancomycin, corresponding to 73% of the total CNS strains identified, while 09 strains (64%) showed resistance to tetracycline and gentamicin, 08 strains (57%) were resistant to neomycin, erythromycin, and chloramphenicol, 07 strains (50%) were resistant to sulfamethoprim, 05 strains (36%) were resistant to linezolid, 03 strains (21%) were resistant to rifampicin, 02 strains (14%) were resistant to ciprofloxacin and cefepime, and 01 strain (7%) was resistant to clindamycin (Table 3).

The multiresistance of CNS strains demonstrated herein is consistent with previous studies on coagulase-negative and coagulase-positive staphylococci that have found several resistant and multiresistant* Staphylococcus aureus* strains in raw milk, meat, and fermented meat products [43].

Surprisingly, the resistance to chloramphenicol is very similar to that estimated for MRCNS strains isolated from human clinical samples. Indeed, there is no direct correlation between the researched features and the origin of the staphylococci strains, since different virulence factors are widespread, such as antibiotic resistance [44], reinforcing the fact that the ability of food matrices strains to produce SE and SE*l* and their multiresistance character must be considered when evaluating the safety hazards of food poisoning.

*S. epidermidis* KF600589.1 and* S. hominis* JX519988.1 showed the highest MAR indexes (0.93 and 0.80, resp.). These strains found in artisanal salami are homologous to strains isolated from human skin microbiota and should be carefully considered among the potential pathogenic staphylococci found in food from animal origins, which can be caused by contamination by poor hygienic conditions during salami manufacturing.

Depending on the conditions, some species of coagulase-negative staphylococci can present health risks, since they have shown resistance to antibiotics of therapeutic importance, such as beta lactams [15]. However, multiresistant strains like* S. carnosus* KJ1862002.1 and* S. xylosus* CP007208.1 and CP008724.1 were intentionally introduced in the food matrix, since they are part of the culture starter used in Italian-salami manufacturing. As previously discussed, food can be a reservoir of multiresistant microorganisms that can spread by the consumption of an apparently safe food. Due to the intensive and indiscriminate use of antibiotics for human and veterinarian therapeutic purposes, multiresistant staphylococci strains are being selected and reproduced in food matrices [45].

The cefoxitin and oxacillin disk diffusion tests are recommended for determining the resistance and susceptibility breakpoint for MRSA surveillance cultures [46]. In this study, there was a good correlation between the disc test zone diameters for oxacillin and cefoxitin (Table 3). The MICs for ampicillin and methicillin/oxacillin/cefoxitin for all susceptible CNS strains were determined by the diffusion disc test, using 0.03 to 2 mg mL^−1^ of each antimicrobial agent.

*S. saprophyticus* HF937252.1 showed an MIC of 0.03 mg mL^−1^ for methicillin, strain* S. carnosus* KJ862002.1,* S. xylosus strains* CP008724.1 and CP007208.1, S*. saprophyticus* JX490122.1,* S. hominis* JX519988.1, and* S. succinus* KC329824.1 were resistant to 0.06 mg mL^−1^ and two strains,* S. saprophyticus* KJ699151.1 and* S. saprophyticus* KJ004623.1, showed an MIC of 0.5 mg mL^−1^ (Table 4). Two of the methicillin-resistant strains,* S. saprophyticus* KJ004623.1 and* S. xylosus* CP007208.1, harbor no enterotoxin gene.

Five strains,* S. saprophyticus* JX490122.1,* S. carnosus* KJ862002.1,* S. xylosus* CP008724.1,* S. xylosus* KF198080.1, and* S. saprophyticus* KJ004623.1, showed an MIC for ampicillin of 0.03 mg mL^−1^, two strains,* S. succinus* KC329824.1 and* S. saprophyticus* KJ699151.1, showed an MIC of 0.25 mg mL^−1^, and* S. hominis* JX519988.1 showed an MIC of 0.5 mg mL^−1^ (Table 4).* S. saprophyticus* KJ004623.1 also showed an MIC of 0.03 mg mL^−1^ but does not harbor enterotoxin genes. It is important to note that most of the CNS strains that showed resistance to ampicillin were also resistant to methicillin, as highlighted by the MIC tests.

Five strains,* S. saprophyticus* HF937252.1,* S. saprophyticus* KJ699151.1,* S. saprophyticus* JX490122.1,* S. saprophyticus* KJ004623.1, and* S. succinus* KC329824.1, showed an MIC for vancomycin of 0.03 mg mL^−1^, one strain,* S. xylosus* CP008724.1, showed an MIC of 0.06 mg mL^−1^, one strain,* S. saprophyticus* EU430992.1, showed an MIC of 0.25 mg mL^−1^, and two strains,* S. xylosus* KF198080.1 and* S. saprophyticus* AB697717.1, showed an MIC of 0.5 mg mL^−1^ (Table 4).

Additionally, five CNS strains,* S. xylosus* CP008724.1,* S. saprophyticus* JX 490122.1,* S. succinus* KC329824.1,* S. saprophyticus* KJ699151.1, and* S. saprophyticus* KJ004623.1, showed resistance to methicillin, as demonstrated by the MIC determinations (Table 4).

Previous studies have demonstrated that linezolid is active against Gram-positive bacteria, including methicillin-resistant staphylococci [47]. In the present study, 05 strains showed linezolid resistance in the disc diffusion test, but it was impossible to establish MIC values for* S. epidermidis* KF600589.1,* Staphylococcus hominis* JX519988.1, and* Staphylococcus carnosus* KJ862002.1. The remaining strains* S. saprophyticus* EU430992.1 and* S. xylosus* CP007208.1 presented an MIC for linezolid of 0.125 mg mL^−1^ and 0.25 mg mL^−1^, respectively.

The MIC of 0.03 mg mL^−1^ for penicillin is in accordance with the previous values estimated for resistant* S. aureus* strains found in several food matrices, such as meat, dairy products, and ready-to-eat food [48].

There is still a lack of information on the antimicrobial resistance of staphylococci strains from food matrices, although they are the most worrisome vehicles of dissemination of antibiotic-resistant pathogens [49].

The high resistance found for salami staphylococci strains can be ascribed to their inappropriate use as growth promoters of antimicrobial agents like oxacillin, vancomycin, chloramphenicol, neomycin, and erythromycin, which are commonly used in veterinary medicine to treat infections [50]. Antimicrobial therapy of infections staphylococci is based on results of susceptibility tests* in vitro* [51]. Methicillin-resistant coagulase-negative* Staphylococcus* spp. found in ready-to-eat products such as meats, fish, and dairy products also offer risks and, although they are not classical food poisoning bacteria, their presence in food offers significant risks to public health due to the possible spread of antibiotic resistance [52].

The standardization of salami quality should include diagnostic methods to screen and quantify the presence of classical and newly described enterotoxins directly in the food matrices as a routine procedure to be conducted by the meat product industry in Brazil.

The safety of salami consumption could also be enhanced by the inclusion of a microbial barrier such as the inclusion of probiotic strains producing natural antibiotics or competitive flora or even the addition of natural bioagents against spoilage or pathogenic microorganisms.

## Figures and Tables

**Table 1:** Primer set for V3 16S rDNA sequencing and PCR/real time RT-PCR tests targeting the classical and newly described staphylococcal enterotoxin genes.

	Primers set and sequences (5′-3′)	Gene	Amplicon (bp)	References
SEA_f_	TTGGAAACGGTTAAAACGAA	*sea*	120	[17]
SEA__r__	GAACCTTCCCATCAAAAACA			

SEB_f_	TCGCATCAAACTGACAAACG	*seb*	478	[17]
SEB_r_	GCAGGTACTCTATAAGTGCC			

SEC_f_	GACATAAAAGCTAGGAATTT	*sec*	257	[17]
SEC_r_	AAATCGGATTAACATTATCC			

SED_f_	CTAGTTTGGTAATATCTCCT	*sed*	317	[17]
SED_r_	TAATGCTATATCTTATAGGG			

SEE_f_	TAGATAAAGTTAAAACAAGC	*see*	170	[17]
SEE_r_	TAACTTACCGTGGACCCTTC			

SEG_f_	TGCTATCGACACACTACAACC	*seg*	704	[18]
SEG_r_	CCAGATTCAAATGCAGAACC			

SEH_f_	CGAAAGCAGAAGATTTACACG	*seh*	495	[18]
SEH_r_	GACCTTTACTTATTTCGCTGTC			

SEI_f_	GACAACAAAACTGTCGAAACTG	*sei*	630	[18]
SEI_r_	CCATATTCTTTGCCTTTACCAG			

SE*l*J_f_	CAGCGATAGCAAAAATGAAACA	*selj*	426	[19]
SE*l*J_r_	TCTAGCGGAACAACAGTTCTGA			

SE*l*M_f_	CCAATTGAAGACCACCAAAG	*selm*	517	[20]
SE*l*M_r_	CTTGTCCTGTTCCAGTATCA			

SE*l*N_f_	ATTGTTCTACATAGCTGCAA	*seln*	682	[20]
SE*l*N_r_	TTGAAAAAACTCTGCTCCCA			

SE*l*O_f_	AGTCAAGTGTAGACCCTATT	*selo*	534	[20]
SE*l*O_r_	TATGCTCCGAATGAGAATGA			

SE*l*K_f_	ATGAATCTTATGATTTAATTTCAGAATCAA	*selk*	545	[21]
SE*l*K_r_	ATTTATATCGTTTCTTTATAAGAAATATCG			

SE*l*Q_f_	GGAAAATACACTTTATATTCACAGTTTCA	*selq*	539	[21]
SE*l*Q_r_	ATTTATTCAGTTTTCTCATATGAAATCTC			

SE*l*R_f_	AATGGCTCTAAAATTGATGG	*selr*	363	[22]
SE*l*R_r_	TCTTGTACCGTAACCGTTTT			

SE*l*U_f_	AATGGCTCTAAAATTGATGG	*selu*	215	[22]
SE*l*U_r_	ATTTGATTTCCATCATGCTC			

TSST-1_f_	ATGGCAGCATCAGCTTGATA	*tstH1*	350	[17]
TSST-1_r_	TTTCCAATAACCACCCGTTT			

16S rDNA_f_	ATA AGA CTG GGA TAA CTT CGG G	*16SrDNA*	500	[23]
16S rDNA_r_	CTT TGA GTT TCA ACC TTG CGG TCG			

f: forward; r: reverse.

**Table 2:** Genotypic and phenotypic characterization of CNS strains from salami.

Strains identification and characterization				
Salami origin	*Staphylococcus* species	Genotypic		Phenotypic
	GenBank accession number and similarity (%)	Presence of enterotoxin genes	mRNA detection	Enterotoxin production (ng mL^−1^)
Commercial	*Staphylococcus *spp. KF135445.1 (96)	*sea*,* seb*	—	—
	*S. carnosus* KJ862002.1 (96)	*sea*,* seb*,* sed*,* seh*,* sei*,and *selj*	—	1.2 ± 0.1
	*S. carnosus *NR116434.1 (98)	*seb*,* sec*,* sed*, and* see*	*see*	0.3
	*S. saprophyticus *AB697717.1 (98)	*seh*,* sei*, and* selm*	*sei*,* selm*	0.3
	*S. saprophyticus *EU430992.1 (99)	*sea*,* seb*,* selm*,* seln*,* selo*,and* tstH1*	*seb*,* selm*, and* selo*	1.3 ± 0.1
	*S. saprophyticus *HQ699510.1 (97)	—	—	—
	*S. saprophyticus *JX490122.1 (99)	*sea*,* seh*	*seh*	0.5 ± 0.1
	*S. saprophyticus* KJ004623.1 (96)	*sec*	—	0.4
	*S. saprophyticus * KJ949606.1 (98)	*sea*,* seb*,* sec*,and* seh*	*sea*,* seb*, and* seh*	—
	*S. sciuri *JX966436.1 (98)	*sed*,* sei*	*sei*	1.0 ± 0.1
	*S. xylosus *AM882700.1 (97)	—	—	—
	*S. xylosus * CP007208.1 (99)	—	—	—
	*S. xylosus *CP008724.1 (98)	*sea*,* tstH1*	*sea*	1.4
	*S. xylosus *KC456590.1 (98)	—	—	—

Artisanal	*S. epidermidis *KF600589.1 (97)	*sec*,* sed*,* see*,* seg*,* seh*,and *sei*	*see*,* sei*	0.7 ± 0.1
	*S. hominis *JX519988.1 (97)	*sea*,* seb*,* sec*,* sed*,* selj*,* selm*,* seln*, and* selo*	*seln*,* selo*	0.9 ± 0.1
	*S. saprophyticus *HF937252.1 (97)	*seb*	*seb*	0.5
	*S. saprophyticus *subsp.* bovis *KJ699151.1 (98)	*sec*,* selm*,* seln*,and* tstH1*	*seln*	0.9 ± 0.1
	*S. succinus *KC329824.1 (99)	*sea*,* seb*,* sec*,* selj*,and* seln*	*seb*,* sec*	1.3
	*S. xylosus *KF198080.1 (97)	*sec*,* selm*,and* tstH1*	—	0.5 ± 0.1

The presence of enterotoxin genes *sea*,* seb*,* sec, sed*,* see*,* seg*,* seh*,* sei*,* selj*,* selk*,* selm*,* seln*,* selo*,* selq*,* selr*,* selu, *and* tstH1* was tested by PCR using the specific set of primers.

*sea*-*see* enterotoxin production was evaluated by immune-sorbent assays (ELISA) using the detection kit RIDASCREEN SET A, B, C, D, E. Values are displayed as the means ± SD of assays performed in duplicate.

mRNA transcripts for all enterotoxin genes were evaluated by real time RT-PCR tests.

CT values are displayed as means ± SD of RT-PCR tests performed in duplicate: *sea*—*S. xylosus* CP008724.1 30 ± 0.4; *seb*—*S. xylosus* CP008724.1 33 ± 2.0; *S. saprophyticus *JX 490122.1 30.2 ± 0.4; *S. saprophyticus *subsp.* bovis *KJ699151.1 32 ± 0.1; *S. xylosus* KF198080.1 36 ± 1.0; and *S. hominis* JX519988.1 34 ± 1.0; *sec*—*S. saprophyticus *subsp.* bovis *KJ699151.1 31 ± 1.1; *see*—*S. saprophyticus *subsp.* bovis *KJ699151.1 30.6 ± 0.5; *S. succinus *KC329824.1 28.5 ± 0.1; and *S. saprophyticus* HF937252.1 32 ± 0.4; *seh—S. xylosus *CP008724.1 31 ± 1.0; *S. saprophyticus *JX966436.1 33.2 ± 0.6; *sei—S. saprophyticus *AB697717.1 30 ± 1.0; *S. saprophyticus *JX966436.1 30.6 ± 1.0; *seln—S. hominis* JX519988.1 30.3 ± 1.0; *S. saprophyticus *EU430992.1 31 ± 0.3; and* selo*—*S. hominis* JX519988.1 32 ± 1.0.

**Table 3:** Multiple resistance to antimicrobial as found in CNS strains from salami.

Salami origin	CNS strains	Antimicrobial agent resistance	Multiple antimicrobial resistance (MAR) index^*∗*^
Commercial	*Staphylococcus *spp. KF135445.1	CIP, CLO, CPM, GEN, NEO, OXA, PEN, SXT, TET, and VAN	0.66
Commercial	*S. carnosus *KJ862002.1	CIP, GEN, LZD, NEO, OXA, SXT, and TET	0.46
Commercial	*S. saprophyticus *AB697717.1	CFO, CLO, ERI, PEN, OXA, TET, and VAN	0.46
Commercial	*S. saprophyticus *EU430992.1	CFO, CLO, ERI, GEN, LZD, NEO, OXA, PEN, SXT, TET, and VAN	0.80
Commercial	*S. saprophyticus *JX490122.1	CFO, CLO, ERI, GEN, NEO, PEN, OXA, SXT, TET, and VAN	0.66
Commercial	*S. saprophyticus *KJ004623.1	CFO, OXA, and PEN	0.20
Commercial	*S. sciuri *JX966436.1	CFO, CLO, GEN, NEO, OXA, PEN, and TET	0.46
Commercial	*S. xylosus *CP007208.1	OXA, LZD, PEN, and VAN	0.26
Commercial	*S. xylosus *CP008724.1	CFO, CLO, ERI, GEN, NEO, OXA, PEN, TET, and VAN	0.60
Artisanal	*S. epidermidis *KF600589.1	CFO, CLI, CLO, CPM, ERI, GEN, LZD, NEO, OXA, PEN, RIF, SXT, and TET	0.93
Artisanal	*S. hominis* JX519988.1	CIP, CPM, CPO, ERI, GEN, LZD, NEO, OXA, PEN, RIF, SXT, and VAN	0.80
Artisanal	*S. saprophyticus *HF937252.1	CFO, OXA, PEN, and SXT	0.26
Artisanal	*S. saprophyticus *KJ699151.1	OXA, PEN, and VAN	0.20
Artisanal	*S. succinus *KC329824.1	CFO, CLO, ERI, GEN, OXA, PEN, RIF, SXT, TET, and VAN	0.66
Artisanal	*S. xylosus *KF198080.1	CFO, CLO, ERI, GEN, NEO, OXA, PEN, TET, and VAN	0.60

^*∗*^The MAR index of an isolate is defined as *a*/*b*, where *a* represents the number of antimicrobials to which the isolate was resistant and *b* represents the number of antimicrobials to which the isolate was subjected.

*S. aureus* strains ATCC WB81 (*sea*), ATCC 13563 (*sed*), and ATCC 27664 (*see*) showing a MAR index of 0.5 and *S. aureus strains *ATCC14458 (*seb*) and ATCCWB72 (*sec*) and *S. xylosus* ATCC 29971 showing a MAR index of 0.3 were used as reference strains.

CPM: cefepime, CFO: cefoxitin, CLO: chloramphenicol, CIP: ciprofloxacin, CLI: clindamycin, ERI: erythromycin, GEN: gentamycin, NEO: neomycin, LZD: linezolid, RIF; rifampicin, TET: tetracycline, OXA; oxacillin, PEN: penicillin, SXT: sulfamethoprim, and VAN: vancomycin.

**Table 4:** Minimal inhibitory concentration (MIC) of compounds used in antimicrobial therapy against staphylococci infections.

Salami origin	GenBank accession number and similarity (%)	MIC mg mL^−1^			
		Methicillin	Ampicillin	Vancomycin	Linezolid
Commercial	*S. carnosus *KJ862002.1	0.06	0.03	—	—
	*S. xylosus *CP008724.1	0.06	0.03	0.06	—
	*S. saprophyticus *JX490122.1	0.06	0.03	0.5	—
	*S. saprophyticus* AB697717.1	—	—	0.5	—
	*S. saprophyticus* EU430992.1	—	—	0.25	0.125
	*S. succinus* KC329824.1	0.06	0.25	0.03	—

Artisanal	*S. hominis.* JX519988.1	0.06	0.5	—	—
	*S. saprophyticus * HF937252.1	0.03	—	—	—
	*S. xylosus* KF198080.1		0.03	0.5	—
	*S. saprophyticus* KJ699151.1	0.5	0.25	0.03	—
	*S. saprophyticus * KJ004623.1	0.5	0.03	0.03	—
	*S. xylosus * CP007208.1	0.06	—	—	0.25

Strains *S. epidermidis *KF600589.1 and *Staphylococcus *spp. KF135445.1 were not susceptible to the antimicrobial concentrations tested in the present study.
